# Text Messaging and Type 1 Diabetes Management: Qualitative Study Exploring Interactions Among Patients and Health Care Professionals

**DOI:** 10.2196/11343

**Published:** 2019-05-10

**Authors:** Francesco Miele, Silvia Clementi, Renzo Gennaro, Ilaria Nicolao, Tiziana Romanelli, Katja Speese, Enrico Maria Piras

**Affiliations:** 1 e-Health Research Unit Bruno Kessler Foundation Trento Italy; 2 Azienda Provinciale per i Servizi Sanitari Diabetes Center Trento Italy; 3 Azienda Provinciale per i Servizi Sanitari Diabetes Center Rovereto Italy

**Keywords:** mHealth, text messaging, type 1 diabetes, diabetes in pregnancy, qualitative research

## Abstract

**Background:**

The diffusion of information and communication technologies (ICTs) in type 1 diabetes (T1D) management has generated a debate on the ways in which ICTs can support the patient-provider relationship. Several studies have focused on text messages. Most of the literature proposes quantitative analysis of the impact of text messaging on the clinical conditions of patients and/or their satisfaction with the technology, while the qualitative studies have focused mainly on patients’ perceptions about strengths and weaknesses of this technology.

**Objective:**

In contrast to past studies, we adopted a qualitative approach for the in-depth examination of patient-health care professionals’ interactions in text messaging.

**Methods:**

The study focused on the use of the Trento Cartella Clinica del Cittadino Diabetes System (TreC-DS), a digital platform with a built-in messaging system, in two diabetes centers, integrating message analysis with interviews with patients and health care professionals. Each center focused on a specific patient profile: the first one focused on pregnant women with T1D and the second one focused on adult patients with poorly controlled diabetes.

**Results:**

The main results of the study were as follows: (1) Health care professionals and patients perceived the messaging system as useful for sharing information (ie, pregnant women for prescriptions and adults with poorly controlled diabetes for advice); (2) The content and communication styles of the two centers differed: in the case of pregnant women, interactions via text messaging were markedly prescriptive, while in the case of adult patients with poorly controlled diabetes, they were conceived as open dialogues; and (3) Conversations were initiated mainly by professionals; in the cases considered, it was mainly the diabetes center that decided whether a messaging conversation was needed.

**Conclusions:**

The results show how the features of interactions of text messaging changed based on the patient profiles in two different centers. In addition, in both diabetes centers that were involved, the system seems to have laid a foundation for a closer relationship between patients and health care professionals.

## Introduction

Type 1 diabetes (T1D) is an autoimmune disease characterized by deficient insulin production in the body that tends to develop in childhood. T1D self-management can be challenging for patients and their relatives, including the following daily tasks [1]: self-monitoring blood glucose levels, managing insulin treatment, observing the symptoms of hypoglycemia (eg, constant hunger, tiredness, and blurred vision), and conducting other activities meant for preventing diabetes complications (eg, self-monitoring foot health and screening for eye complications).

The diffusion of information and communication technologies (ICTs) in health care has generated a polycentric debate on how ICTs can support patients with diabetes, providing services that empower them in self-management (eg, electronic logbooks or reminder functions for medications), enable communication with health care professionals (eg, messaging systems and rule-based alarms), and offer information on self-management (eg, tutorials for blood glucose tests). In particular, this debate has addressed issues such as the clinical impact of ICTs [2-9], the effects of ICTs on the patient-professional relationship [10,11], and their consequences on the workload of hospital staff [12-15].

The existence of a specific strand of studies on ICTs and diabetes is due to some characteristics of this illness, such as the significant workload required of patients and the vital role of patient education [16]. For this reason, although the use of messaging systems in health care is a topic already investigated with regard to other conditions [17-21], various contributors have focused explicitly on the role of text messaging in supporting and educating patients with diabetes. These studies often focus on the impact of text messaging on clinical outcomes and on the self-care capabilities of diabetic patients [22-27]. Other researchers have paid attention to patients’ perceptions of the usefulness of text messaging [28-31], privileging quantitative techniques to assess users’ satisfaction. Finally, another group of studies has adopted qualitative techniques to explore patients’ perceptions regarding strengths and weaknesses of text messaging [32-37]. These works underline that text messaging is perceived by patients as a tool that is useful for resolving nonurgent issues with health care professionals [32], accessing and managing their own clinical data [37], receiving information and analytics for self-management [33,36], and feeling monitored [34,35]. Qualitative studies have also performed in-depth analysis of the factors perceived by patients that discourage the use of text messaging, such as their unfamiliarity with digital devices [21] or slow responsiveness of professionals [24].

As Holtz and Laukner argue [38], studies on text messaging have several limitations. The reports of provider interactions with diabetic patients through text messaging are limited and poorly documented, while health care professionals’ perceived usefulness and the integration of mobile apps in organizational workflows are underinvestigated. This work intends to contribute to fill in the first gap, adopting a qualitative approach for documenting and examining, in depth, the interactions of patients and health care professionals via text messaging.

We consider the case of the Trento Cartella Clinica del Cittadino Diabetes System (TreC-DS), a digital platform with a built-in messaging system that supports communication between health care organizations and T1D patients. The study investigates the use of the messaging system in two diabetes centers in the Province of Trento, Italy. These two diabetes centers used the TreC-DS to support patients who would benefit from strict monitoring: pregnant women with T1D and adults with poorly controlled diabetes.

In the next section of this paper, we introduce the TreC-DS and the methods used to gather and analyze data. The findings, preceded by an overview analysis of the frequency of message exchanges, are organized into two subsections, one for each diabetes center. We conclude with some final remarks about the use of text messaging in T1D management and discuss limitations to this study.

## Methods

### The TreC Diabetes System

The Trento Cartella Clinica del Cittadino (TreC), a citizen-controlled clinical record system, was introduced to the Province of Trento in 2010 with the aim of empowering Italian citizens to manage their own health, facilitating their communication with health care institutions and the management of health information [39]. The platform has two TreC services: *basic* and *composite*. The former consists of data management and other common Web-based functions, and the latter includes higher integrated functions, such as a structured health diary and monitoring tools for specific pathologies [40].

**Figure 1:**
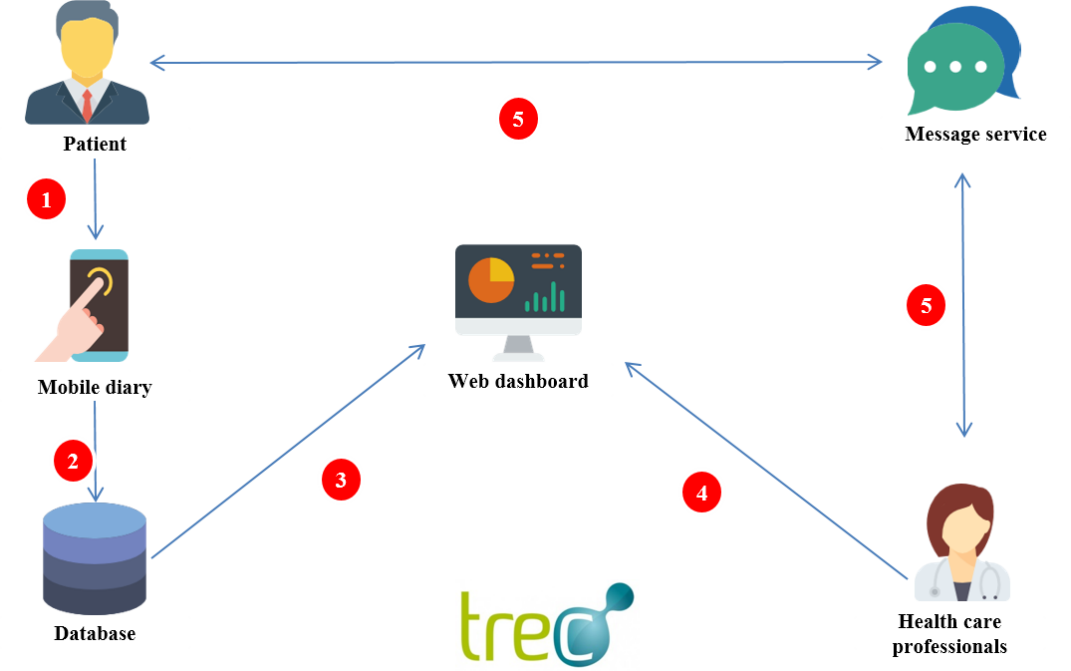
A conceptual model of diabetes patient home monitoring: (1) daily diary compilation; (2) data stored in a central database; (3) data displayed on a dashboard; (4) health care professionals from the diabetes center access patient dashboards to evaluate patient problems; (5) conversation between patient and professionals about diabetes management.

The TreC-DS was developed to deliver mHealth services to citizens with T1D and to diabetes centers (see Figure 1). A mobile phone app enabled patients to keep track of all their diabetes information (eg, measurements, therapy, symptoms, and diet); it also included functions to support decision making (ie, a carbohydrate-count feature, a bolus calculator, graphs, and trend-tracking indexes). Through a Web-based dashboard, health care professionals (ie, doctors and nurses) could monitor patients’ data remotely. Finally, the platform had a built-in messaging system that worked as a secure text-messaging service between patients and professionals.

### Clinical Trial

This work is a part of a trial aimed at quantifying the effectiveness and acceptability of the TreC-DS for T1D patients. The trial was approved by the Research Ethics Committee of the Health Authority of the Autonomous Province of Trento, and written consent was obtained from the participants. The consent forms were included among other signed documents upon registration of the clinical trial at Ministero della Salute Direzione Generale dei Dispositivi Medici del servizio Farmaceutico e della Sicurezza delle Cure (DGDFSC) (trial number 0032830-P-22/04/2014).

The trial involved three diabetes centers in the Province of Trento. Each center focused on a particular patient profile and involved 10-15 patients:

1. The *Diabetes Center-Pediatrics* selected 15 children and adolescents who had poorly controlled diabetes, had a recent diagnosis, or used an insulin pump.
2. The *Diabetes Center-Adults* chose 15 patients with poorly controlled diabetes.
3. The *Diabetes Center-Pregnancy* selected 10 pregnant women with pre-existing T1D.

During the definition of the clinical trial protocol, these diabetes centers targeted patient profiles that would, according to the involved health care professionals, benefit from the stricter monitoring and frequent reminders about correct disease management that the platform could provide.

While the evaluation of the trial focused on the self-management practices of patients, changes in organizational practices arising from the introduction of the new technology to the diabetes centers and changes in the patient-professional relationship were also examined [11]. Here we present part of the results yielded from this research project, focusing on the use of the messaging system utilized by the Diabetes Center-Adults and Diabetes Center-Pregnancy, which were chosen for their frequency of use, integration of analyses of these messages, and interviews between health care professionals and patients. Characteristics of patients from these centers are summarized in Table 1.

### Data Gathering

The use of the TreC-DS was explored through the analysis of text messages exchanged between patients and professionals via the system. The messages consisted of analyses of patient data, as well as inquiries and comments on insulin therapy, diet, and diabetes self-management. We analyzed 396 conversations within message threads exchanged between the patients and centers.

After a preliminary analysis of message exchanges, we conducted semistructured interviews with health care professionals and patients (see Textbox 1).

We interviewed all patients selected by the Diabetes Center-Pregnancy, except one that did not consent to the interview, as well as the first 8 patients to give their availability who were selected by the Diabetes Center-Adult with the aim to have comparable numbers of interviewed patients from each center. Patients were interviewed at home. Interviews lasted 45-60 minutes and were transcribed verbatim. All health care professionals involved in the clinical trial were interviewed. The main topics of the interviews are summarized in Table 2.

**Table 1:** Characteristics of patients and their use of text messaging.

Diabetes center and patient number		Gender	Age in years	Number of conversations with professionals via text messaging	Interviewed
**Pregnancy**						
	1	Female	38	17	Yes
	2	Female	32	16	Yes
	3	Female	26	4	No
	4	Female	37	9	Yes
	5	Female	38	6	Yes
	6	Female	28	20	Yes
	7	Female	32	9	Yes
	8	Female	36	12	Yes
	9	Female	22	21	Yes
	10	Female	33	9	Yes
**Adults**						
	1	Male	39	27	Yes
	2	Male	23	13	Yes
	3	Male	41	5	No
	4	Male	40	15	Yes
	5	Male	49	17	Yes
	6	Female	27	18	No
	7	Female	37	17	Yes
	8	Female	20	23	Yes
	9	Male	32	22	No
	10	Male	50	25	Yes
	11	Female	50	19	Yes
	12	Male	29	25	No
	13	Male	42	36	No
	14	Male	21	4	No
	15	Male	31	7	No

**Target populations involved in the interviews.**

Diabetes center and Interview participants

- Adults
  - 8 patients
  - 2 doctors (diabetologists)
  - 1 nurse
- Pregnancy
  - 9 patients
  - 1 doctor (diabetologist)
  - 1 nurse

**Table 2:** Interview guide.

Interview topics	Interview subtopics	
	Patients	Health care professionals
Patient-professional relationship (before TreC-DS^a^)	Disease onset Self-management practices Use of self-tracked data	Education at onset and during clinical encounters Use of patient-tracked data
Use of TreC-DS	Collection and data analysis Use of text messaging Relationship between the use of TreC-DS and other care practices Changes in relationship with professionals	Use of patient-tracked data Use of text messaging Relationship between the use of TreC-DS and other organizational practices Changes in relationship with patients
General evaluation of TreC-DS	Usefulness of TreC-DS Intention to use TreC-DS after the trial	Usefulness of TreC-DS Intention to use TreC-DS after the trial

^a^TreC-DS: Trento Cartella Clinica del Cittadino Diabetes System.

The interviews focused on reconstructing the ways in which patients and professionals used the different features of the TreC-DS, including text messaging, during the trial. In addition, during the interviews we investigated elements not directly connected with the use of the system. This latter focus was investigated, since it is assumed that the ways in which people use and incorporate a new technology into their daily lives are shaped by social circumstances, such as patients' attitudes toward their illnesses, pre-existing relationships and practices, and emerging social representations about the new technology [41].

### Data Analysis

#### Overview

Data gathered were coded using template analysis, through which “the researcher produces a list of codes (‘template’) representing themes identified in their textual data. Some of these will usually be defined a priori, but they will be modified and added to as the researcher reads and interprets the texts” [42]. Preliminary categories based on the interview outline and literature analysis were used to segment the texts [32-38].

#### Message Analysis

At the end of message analysis, we created two general categories. The first category was labelled “communication style of the health care professional-patient interaction,” which was concerned with how much the interactions between professionals and patients were prescriptive or open. The second one was labelled “contents of the health care professional-patient interaction,” which was concerned with topics of the text messages. The contents of the conversations (ie, the message threads) were subdivided into the following subcategories:

1. Therapy: management of glycemia through insulin therapy.
2. Diet: management of glycemia through food intake.
3. Education: general rules for self-management.
4. Motivation: reinforce adherence to treatment.
5. Context: gathering information regarding patients’ daily lives (eg, exceptional events and comorbidities) that could affect diabetes management.
6. Technical issues: problems that emerged in data tracking.

Conversations often covered multiple topics at once. In these cases, we coded the single conversation more than once.

#### Interview Analysis

Interviews were also analyzed using template analysis. The purpose of this second step of analysis was to explore patients’ and professionals’ use and social representations of the TreC-DS, enriching the content of the categories described above. During the analysis of the interviews, another category emerged, which was labelled “representations about text messaging.” This last category concerned the meaning given to text messaging by both patients and health care professionals (eg, how the system improved doctor-patient communication or self-care capabilities).

## Results

### Overview

We conducted a preliminary analysis of the frequency with which the patients and diabetes centers used the TreC-DS messaging feature during the trial. We focused on two topics: the direction of each conversation (ie, who initiated the interaction) and the shared content of the messages using the categorization described above. Regarding the direction of conversation (see Table 3), there were no remarkable differences between the messages from the two diabetes centers. In both cases, the majority of the conversations were initiated by the center; the percentage of center-initiated messages was somewhat higher among the adult patients with poorly controlled diabetes (231/273, 84.6%) than it was among the pregnant patients with diabetes (100/123, 81.3%).

**Table 3:** Direction of text-message conversations.

Diabetes center	Conversations, N	Direction of conversation, n (%)	
		Diabetes center to patient	Patient to diabetes center
Pregnancy	123	100 (81.3)	23 (18.7)
Adults	273	231 (84.6)	42 (15.4)

**Table 4:** Text-message conversation content.

Diabetes center	Conversation content, n (%)					
	Education	Motivation	Insulin therapy	Diet	External context	Technical issues
Pregnancy (N=156 conversations)	0 (0)	14 (9.0)	80 (51.3)	22 (14.1)	25 (16.0)	15 (9.6)
Adults (N=341 conversations)	24 (7.0)	104 (30.5)	94 (27.6)	27 (7.9)	25 (7.3)	67 (19.6)

In contrast, if we consider the content of the conversations (see Table 4), many differences can be observed between the Diabetes Center-Pregnancy and Diabetes Center-Adults groups. In the Diabetes Center-Pregnancy group, more than half of the content (80/156, 51.3%) of the conversations focused on therapy. This focus can be explained by the pregnant women’s need to keep their glucose values within a fixed range by the center. Exchanges regarding these patients’ external context (25/156, 16.0%) and diet (22/156, 14.1%) were quite frequent, likely for the same reason. In contrast, in the Diabetes Center-Adults group, the conversations were mainly comprised of motivational content (104/341, 30.5%) aimed at pushing patients with poorly controlled diabetes to achieve good results in the self-management of blood glucose levels. Messages concerning insulin therapy (94/341, 27.6%) and technical issues (64/341, 19.6%) were also frequent; the high percentage of technical issues is probably due to the sporadic access of adult patients to their hospital ward (ie, one or two face-to-face visits in a year). Drawing on our qualitative analysis of text-message exchanges and interviews, we will illustrate the ways in which the patients and health care professionals used and represented the built-in messaging system.

### Diabetes in Pregnancy

T1D has several potential negative effects on the fetus, including overgrowth [43], the development of congenital malformations [44], and premature death [45]. To avoid these negative events, doctors generally recommend strict control over blood glucose levels and over the patient’s lifestyle.

The goal of pregnancy was so important that I began to take many more medical exams and check-ups. [Before the pregnancy] I spent two or three years under strict control and, in the end, I entered the pregnancy with good blood sugar levels.

Patient 1, Diabetes Center-Pregnancy

I’m always scared to do what comes into my head arbitrarily, even when I know these injections can be managed autonomously.

Patient 7, Diabetes Center-Pregnancy

A stricter monitoring of clinical parameters may begin before the pregnancy and continue until childbirth, during which time women may partially renounce their autonomy. The temporary loss of autonomy is justified by both the clinical risks and the loss of relevance of self-management skills due to the physiological changes related to pregnancy. Therefore, the relationship between patients and their diabetes center changes dramatically. While health care professionals shift from an approach based on the empowerment of patients to a more prescriptive one, patients usually accept, and often openly appreciate, this change. In this context, the TreC-DS is represented by both professionals and patients as a technology that is useful for supporting diabetes center-patient communication remotely during a stage of illness in which communication is vital.

Without doubt, it [the use of TreC-DS] is a time-consuming activity...However, the quality of care has changed. If we see the patient after two weeks with the system, it is not like it is when we see a patient after two weeks without the system. It is resuming something that, actually, has never stopped.

Nurse, Diabetes Center-Pregnancy

The relationship with the ward was not as strong as it was previously: the visits, instead of being held once a week, were held twice a month. Nevertheless, weekly advice about therapy still arrived. I felt well monitored, and it [the TreC-DS] has been a great tool.

Patient 4, Diabetes Center-Pregnancy

The TreC-DS was represented by both patients and health care professionals as a system that improves the quality of care. By using it, the diabetes center could monitor patients strictly, request additional information, send prescriptions, and have more-focused conversations during the face-to-face visits with patients, having already acquired information about the patient’s health status from the TreC-DS. For the patients, the system improved the quality of care and reduced the frequency of face-to-face visits, while ensuring continuous interaction with the diabetic center by receiving prescriptions and advice through the system.

The general representations of the system seemed to affect the communication styles of the messages, as shown in the conversation below between Patient 5 and the Diabetes Center-Pregnancy:

Hello. We checked your glucose values, and we noticed that they tend to increase both before dinner and when you wake up. In the afternoon, when you have a snack, try to increase your intake of NovoRapid [short-acting insulin] to 3 units; in the evening, you should increase your Levemir dosage [long-acting insulin] to 17 units. See you soon!

Diabetes Center-Pregnancy, message sent to patient

Good morning. Lately when I wake up, my glucose values are in the 120-138 range before breakfast. I usually take 6 units of Levemir at 11 p.m. In your opinion, do I need to increase my insulin dosage from 6 to 7 units? Thanks, and see you soon!

Patient 5, question posed to Diabetes Center-Pregnancy

Good morning. Your insulin treatment plan has been changed in the following manner: take 4 units of NovoRapid at breakfast, 7 units at lunch, and 7 at dinner; take 8 units of Levemir before bed.

Diabetes Center-Pregnancy, in response to patient’s question

The reported messages, although they contain different directions, are characterized by a prescriptive communication style. The patients with “bad” glycemic values, whether directly observed by health care professionals or reported by the patients, received therapeutic prescriptions, as shown in the conversation below between Patient 7 and the Diabetes Center-Pregnancy:

Good morning. I need information about something that happened this afternoon: 2 hours after lunch, at 5 p.m., I measured my glycemic value at 188. Therefore, I walked for an hour and a half. Then I took a vitamin supplement and two candies labelled “without sugar.” At 8 p.m., my glycemic value was 197! I do not understand. Why have my blood sugar values not decreased? In 3 hours, my values have remained high, even though I walked a lot.

Patient 7, question posed to Diabetes Center-Pregnancy

Hello. The walk was a good idea. You should always check the carbohydrates indicated on packages of food such as candy. The inscription “without sugar” means that the candy does not contain any added sugar, but it still does contain sugar.

Diabetes Center-Pregnancy, in response to patient’s question

Nutrition was an area of autonomy that was rarely touched. However, the professionals did discuss the sugars contained in foods and how to handle them, whether through insulin therapy or physical activity. The communication style was less prescriptive on this topic, but the advice of the ward was generally considered authoritative.

Hello! Maybe you did not read the message written by the doctor. We are waiting to receive your blood sugar values, including those from the last few days.

Diabetes Center-Pregnancy

Given the high attention of the diabetes center on diabetic women in pregnancy, messages similar to the one above were quite common. Through these messages, the center aimed at continuous monitoring of patients; however, monitoring was only accepted for the duration of pregnancy.

No! After the pregnancy, I would not use the system every day. Right now, I have a goal I care about, and I am alert to everything. But after the pregnancy, I would feel really sick [using it].

Patient 10, Diabetes Center-Pregnancy

The women with diabetes who were interviewed agreed that continuous use of the TreC-DS would be unacceptable and too onerous after pregnancy. Therefore, the suspension of diabetes self-management was interpreted as temporary. Some patients claimed that if they were not pregnant, they would not use the system at all, while others favored a more limited use of the system. For the latter, the system was represented as useful for sending specific data to the diabetes center, allowing them to receive feedback from professionals about their self-management strategies.

### Poorly Controlled Diabetes

In the medium-to-long term, patients with poorly controlled diabetes may develop serious health issues, such as cardiovascular diseases [46,47], retinopathies [48,49], or renal diseases [50,51]. Therefore, health care professionals have included adults with a history of poor self-management in the study, with the aim of monitoring them remotely.

My history with diabetes started out badly. When I was hospitalized, I was barely saved. From that moment on, I began to have practical issues with diabetes management: my life is fast-paced and stressful, and for a long time, I did not accept my illness. I approached the Diabetes Centre of Trento, which I frequented for some time, but then I abandoned it. Then, several years ago, I realized that I had to regain control over my life.

Patient 7, Diabetes Center-Adults

The above excerpt exemplifies why many patients experience difficulties accepting their illness and reconciling it with their social and work lives. Consequently, friction often arises between these patients and health care organizations. The TreC-DS was adopted by patients who had tried several times to improve their self-management capabilities after being alerted by a diabetes center that, in the future, their health conditions could become worse. The patients’ histories were marked by various failures, and the system was an opportunity to regain control over their condition.

Over the last few years, diabetes management has changed dramatically. We have to monitor our patients, explaining to them, “Be careful: for injections, you now have to use a needle.” Other times, it can happen that [we need to intervene].

Doctor, Diabetes Center-Adults

I greatly appreciated that clinicians and nurses were monitoring me...[Name of nurse] is very active; she gives me suggestions and, if I am in doubt, she answers my questions...and she does so on the basis of the values that I have input into the system!

Patient 5, Diabetes Center-Adults

Both for professionals and patients, the TreC-DS was useful for improving diabetes center-patient relationships, renewing and expanding the self-management skills of patients. From the professionals’ perspective, poor control of diabetes occurs largely because patients have developed a bad relationship with their illness and because they have gaps in knowledge of diabetes. Given these assumptions, the system is useful for monitoring patients, acquiring information about their self-management capabilities, and giving them information and advice.

Yet the patients emphasized that through the TreC-DS, they felt constantly monitored, receiving messages with useful “tricks” for improving their self-management capabilities and feedback on trends in their blood glucose levels. For many of these patients, the system made face-to-face visits less necessary, reducing their frequency. After the introduction of the TreC-DS, patients rapidly shifted from a state characterized by poorly controlled diabetes to one characterized by daily interaction with a diabetes center, receiving feedback and advice.

While you had the flu, you managed your blood sugar values excellently! I hope that you’ll get well so that you can celebrate the New Year. Happy New Year! Goodbye.

Diabetes Center-Adults

When the nurse writes me a message saying “Well done!” I like it, even if I don’t know who is talking to me. I don’t care; I know that, in any case, it is someone knowledgeable.

Patient 9, Diabetes Center-Adults

The system was useful for patients to receive feedback on their self-management skills. As noted previously, the majority of the conversations contained motivational messages sent by the health care department, with the aim of praising patients for the achievement of good blood glucose values. Another aim was to reassure patients who did not reach their clinical goals. The health care providers seemed interested in being noticeable to patients, pointing out to patients that they were monitoring them remotely and encouraging them to track their values daily. It was difficult for these patients to learn self-management skills; for the professionals, the use of the TreC-DS helped to support these patients on their journey.

Hi! Do you know “the rule of 15” for hypoglycemia management? When you have lowblood glucose levels, you have to adjust them with 15 grams of sugar, and after 15 minutes, you have to recheck your blood sugar values: if they are under 100, you have to take 15 grams of sugar again...

Diabetes Center-Adults

Encouraging messages were often followed by educational ones that provided general rules about diabetes management, such as in cases of incorrect patient behaviors observed remotely by health care professionals. Unlike the observations in the case of diabetes in pregnancy, the goal was not to drive patients remotely through prescriptive guidelines, but rather to provide them with advice and rules meant to improve their self-management skills. Consequently, messages concerning insulin therapy and diet took the form of suggestions that the patient could accept, refuse, or follow partially.

Hi! Have you checked your blood sugar values? I noticed that you are not taking the 8 units of Humalog [short-acting insulin] I prescribed for you because you need more insulin. Try taking 10 units of Humalog in the morning, 10 units in the middle of the day, and 10 units in the evening. Try this out for a few days, and let me know how it goes.

Diabetes Center-Adults, message to patient

Okay, but for the middle of the day, I’d like to stay at 8 units of Humalog because I don’t eat much.

Patient 6, message to Diabetes Center-Adults

Okay. This is up to you.

Diabetes Center-Adults, in response to patient’s message

The above conversation reveals that the patient only partially accepted the nurse’s recommendations on modifying her insulin therapy, maintaining that before lunch, she would preserve the pre-existing plan. The suggestions provided by the diabetes center, following the analysis of data tracked in the TreC-DS, were renegotiated by patients in light of their eating habits and living conditions.

## Discussion

### Principal Findings

This paper analyzed how the TreC-DS, an mHealth platform with a built-in messaging system, was used by two diabetes centers to assist two types of patients with T1D. The main findings of this study are summarized in Table 5.

### Sharing Information for Resolving Different Problems

Health care professionals and patients thought of the messaging system and the overall platform as useful for sharing information. Perceptions about text messaging were strongly influenced by the patients’ histories and by the ways in which professionals and the patients themselves interpreted the patients’ needs. On the one hand, for the women with T1D, pregnancy was a tough decision, as these patients were informed about the need for their diabetes to be under strict control during pregnancy; on the other hand, the adults with poorly controlled diabetes had a history of failure in diabetes management, and they gradually became aware of the clinical risks of this situation. Consequently, in the diabetes centers, the same system was considered a useful tool to address very different problems.

### Constructing a Closer Relationship With the Patient

ICTs have long been associated with the standardization and depersonalization of patient-professional relationships [52]. Several recent studies, however, underlined how ICTs can foster more intimate relationships, where professionals better understand patients’ clinical and emotional conditions and can provide personalized interventions [53,54]. Similarly, our results show how through a messaging system, a hospital department can continuously observe patients’ data, guiding patients remotely in the management of diabetes. Despite some differences in the use of text messaging, in both diabetes centers, the messaging system was used by professionals to construct a closer relationship with patients and guide them to avoid clinical complications.

**Table 5:** Patient-health care professional interactions using text messaging.

Diabetes center	Content of message exchanges	Communication styles	Social representations about text messaging
Pregnancy	Most of the messages were about therapy (80/156, 51.3%).	Interactions were initiated mainly by professionals and were markedly prescriptive.	Useful for transmitting prescriptions from diabetes centers to patients. Face-to-face visits decreased and became more focused on patients’ data.
Adults	Most of the messages were about motivation (104/341, 30.5%) and therapy (94/341, 27.6%).	Interactions were initiated mainly by professionals and were conceived as open dialogues.	Useful for empowering patients’ self-management skills. Face-to-face visits decreased.

### Emerging Asymmetrical Interactions

In our study, we showed how the conversations were mainly initiated by the health care professionals. Most likely, the shared perceptions of the system’s use, interpreted from both sides as useful for transmitting information from professionals to patients, laid a foundation for asymmetrical interactions in which professionals analyze the data input by the patients. Clinicians and administrative staff often express concerns about using telemonitoring technologies because of the possible increase in patient requests [12-15]; however, in the considered cases, it is mainly the diabetes center that decides if a text message conversation is necessary, choosing when and how to begin a conversation with a patient.

### Limitations and Future Work

This study aims to break new ground in the analysis on patient-provider relationships emerging from text-message exchanges. However, it suffers from some limitations that should be addressed by future research. Firstly, the study involved only two target groups of patients with TD1. More research is needed regarding the use of text messaging by other target groups of diabetic patients with different monitoring needs and different socioeconomic backgrounds. Secondly, the number of patients, the time frame of the trial, and, consequently, the workload are limited. The overall positive perceptions by health care professionals about text messaging should be reassessed in the prolonged care of a larger set of users. Thirdly, our work focused on patient-health care professional interactions using text messaging without a systematic comparison of these interactions in the absence of text messaging. The lack of a baseline impeded the ability to make specific considerations regarding the consequences of text messaging on the workflow of the health care professionals. Future research on this topic would benefit from an analysis combining observational methods and performance metrics (eg, frequency of visits and the time spent by professionals for each patient) before and after the introduction of the technology.

### Conclusions

In recent years, a debate has emerged on the role of ICTs in diabetes management. Some studies have focused on text messages. Most of the literature proposes quantitative analysis of the impact of text messaging on the clinical conditions of patients and/or their satisfaction with the technology. Qualitative studies have focused mainly on patients’ perceptions about strengths and weaknesses of this technology. In contrast, we used qualitative techniques for documenting in-depth, patient-health care professional interactions using text messaging, combining message analysis and semistructured interviews. The results show how the features of interactions and perceptions of text messaging changed based on the patient profiles in two different centers. In addition, in both diabetes centers considered, the system seems to have laid a foundation for a closer relationship between patients and health care professionals.

## Abbreviations

DGDFSC: Direzione Generale dei Dispositivi Medici del servizio Farmaceutico e della Sicurezza delle Cure
ICT: information and communication technology
T1D: type 1 diabetes
TreC: Trento Cartella Clinica del Cittadino
TreC-DS: Trento Cartella Clinica del Cittadino Diabetes System
